# A physiologically based hypothesis for learning proprioception and in approximating inverse kinematics

**DOI:** 10.14814/phy2.12774

**Published:** 2016-05-24

**Authors:** Matt Simkins

**Affiliations:** ^1^ MEMM Department, California State UniversityChicoCalifornia

**Keywords:** Inverse kinematics, position gradients, synergy, virtual points

## Abstract

A long‐standing problem in muscle control is the “curse of dimensionality”. In part, this problem relates to the fact that coordinated movement is only achieved through the simultaneous contraction and extension of multitude muscles to specific lengths. Couched in robotics terms, the problem includes the determination of forward and inverse kinematics. Of the many neurophysiological discoveries in cortex is the existence of position gradients. Geometrically, position gradients are described by planes in Euclidean space whereby neuronal activity increases as the hand approaches locations that lie in a plane. This work demonstrates that position gradients, when coupled with known physiology in the spinal cord, allows for a way to approximate proprioception (forward kinematics) and to specify muscle lengths for goal‐directed postures (inverse kinematics). Moreover, position gradients provide a means to learn and adjust kinematics as animals learn to move and grow. This hypothesis is demonstrated using computer simulation of a human arm. Finally, experimental predictions are described that might confirm or falsify the hypothesis.

## Introduction

Proprioception is the sense of one's kinematics. This includes a high‐level, cortical understanding of posture, as well as a low‐level representation of muscle lengths, joint angles, and their relation to Euclidean space. This low‐level aspect of proprioception suggests the existence of a forward kinematic model. Forward kinematics determines position and orientation of the hand given some combination of joint angles, or equivalently, muscle lengths. Put another way, forward kinematics maps muscle lengths in “muscle space” (Kakei et al. 1999) to Cartesian coordinates of the hand in Euclidean space. Notwithstanding, the human arm has proprioceptive receptors in the joints and in the muscles (muscle spindles). These receptors are capable of sensing joint angles and muscle lengths, respectively. Muscle spindles play a predominant role in proprioception (Ferrell et al. 1987). Goal‐directed postures or inverse kinematics, considers kinematics in the opposite direction. Inverse kinematics relates hand locations in Euclidean space to muscle lengths in muscle space. Not only is the mapping from Euclidean coordinates to joint angles is nonlinear, the mapping from joint angles to muscle lengths is also nonlinear. In the case of manipulators that have kinematic redundancies, such as the human arm, hand locations maps to an infinite number of joint angles and muscle lengths.

Current motor theory posits that movements are coded dynamically. Given that activity in the motor cortex is known to encode for velocity (Ashe and Georgopoulos 1994; Moran and Schwartz 1999; Paninski et al. 2004), and force (Taira et al. 1996; Sergio and Kalaska 1998; Li et al. 2001), that assumption appears sound. Muscle synergies (Sherrington 1947; d’ Avella and Bizzi 2005; Cheung et al. 2009; Tresch and Jarc 2009), or similarly motor primitives (Thoroughman and Shadmehr 2000; Flash and Hochner 2005; Gizzi et al. 2010), provide an attractive solution for multijoint movements. According to this view, the central nervous system (CNS) sends a volley of time‐varying activations to various muscle groups in order to achieve motion. Without doubt, such a scheme could generate trajectories, torques, and forces. However, from the perspective of accurate positioning of the hand or even shaping trajectories, muscle synergies, by themselves, are unworkable without a kinematic model of some kind. Lacking kinematics, a separate muscle synergy is needed to move the hand from every conceivable start location to every conceivable end location. Moreover, impedances or muscle fatigue would result in significant and cumulative position errors. One might argue that muscle synergies are workable provided that they incorporate proprioceptive afferents for start and end conditions (Bizzi and Abend 1985), but this is tantamount to saying that a kinematic models exists.

It is known that some cells in the motor cortex encode for a particular direction of motion. Activities of populations of motor cortical neurons encode a population vector that points in the direction of intended movement (Kettner et al. 1988). Population vectors were used to spectacular effect in controlling robotic arms in real time using neuronal activity in the motor cortex (Wessberg et al. 2000; Velliste et al. 2008; Vato et al. 2012). No doubt, such work has huge potential in terms of prosthetics and brain‐controlled interfaces (Schwartz et al. 2006). However, even though population vectors allow a robot to accurately predict an animal's intended movement, this is only accomplished using an implicit inverse kinematic model for the robot's kinematics. The animal's ability to control its own skeletal muscles remains unresolved. It might be argued that the animal's CNS simply calculates kinematics in an analogous way to the robot. However, there exist no known neurological mechanisms that approximate the coordinate frame transformations used in robotics. Moreover, calculating inverse kinematics using conventional methods in robotics proves problematic when applied to complicated systems such as the musculature of the human arm (Full and Koditschek 1999). In this sense, the problem of calculating kinematics touches on aspects of the degrees of freedom problem (Bernstein 1967; Arimoto et al. 2005), or on the “curse of dimensionality” (Full and Koditschek 1999).

A complete theory of motor control must include kinematics. This statement derives from the inescapable truth that hand positions and arm postures are inextricably determined by muscle lengths, muscle attachment points, and skeletal dimensions. Kinematics captures the geometry of problems.

A physiologically based model for calculating kinematics is proposed. This hypothesis is not presented as an overarching model that describes all aspects of muscle control, or even all aspects of kinematics. Problems such as kinematic redundancy resolution, orientation, trajectory shaping, force compensation, muscle contraction dynamics, and so on, are out of scope. Rather, the “virtual points” hypothesis is presented as a kinematic bridge between high‐level cortical processing and low‐level control of muscles. In order to validate the hypothesis, at least in principle, a model that describes virtual points was applied to the human arm and simulated on a computer.

## Methods

### Virtual points hypothesis – spatial representations

The concept of muscle synergy has different meanings in the literature, but it is often described in terms of muscle innervations or forces (Thoroughman and Shadmehr 2000; Cheung et al. 2005; Ting and Macpherson 2005; Danna‐Dos‐Santos et al. 2009). For these purposes, muscle synergies are described in the context of kinematics, which by definition, does not involve forces. Therefore, let us consider a different type of synergy called a muscle length synergy (MLS). An MLS is defined as a combination of muscle lengths for a given posture of the arm. The human arm has kinematic redundancies (not to be confused with redundant muscles). That is, for a given hand location, the arm might assume a range of different postures (Scholz and Schöner 1999). The movements being simulated will restrict the elbow and hand to move in a plane so that kinematic redundancies are not permitted.

In a sense, the musculoskeletal system constitutes a physical embodiment of a kinematic calculator. For example, one might imagine adjusting arm muscle lengths. Without having to calculate the resulting posture, the forward kinematic solution is essentially available by simply observing the physical hand location. Conversely, one might imagine moving the hand to some location and measuring the associated muscle lengths. Thus, the muscle lengths are also available without having to calculate inverse kinematics. The virtual points hypothesis relies on the fact that the muscle lengths associated with a given hand location, whatever those lengths happens to be, is a particular inverse kinematic solution for that location. Thus, if the hand were to move through space while being observed, the observer (or CNS) might record the coordinates in Euclidian space along with the corresponding set of muscle lengths. Indeed, learning kinematics does seem to depend heavily on visual feedback (Wolpert et al. 1995). In an overly simplistic model, a hand location is accomplished by simply fetching the needed set of muscle lengths from a lookup table. The muscles would then change in length until the specified muscle lengths are achieved and a stable posture is obtained (Feldman 1966; McIntyre and Bizzi 1993). However, there is a problem. For the hand to span continuous Euclidian space, an infinite number of recorded coordinate points are required. Thus, for the CNS to employ such a strategy, an infinite amount of memory is required. Therefore, it is assumed that only a finite set of MLS's are retained. This set is denoted as a muscle length synergy matrix, **S**.

Due to the finite information storage capacity in the CNS, it might seem that the hand would be restricted to move to specific locations in task space. However, this limitation is surmountable using interpolation. In terms of spaces, interpolation along a line requires two independent pieces of information. Interpolation along a surface requires three independent pieces of information. Interpolation along a volume requires four independent pieces of information. Given that the hand moves in 3D Euclidean space, it is assumed that interpolation will require four independent pieces of information. Another generalization relates to the dimensionality of coordinates. For a line, points describe coordinates. For a surface, coordinates relate to independent or nonparallel lines along the surface. The intersection of any two lines defines a coordinate point. For a volume, coordinates relate to independent planes. The intersection of any three nonparallel planes describes a coordinate point. Again, because the hand moves in 3D Euclidean space, it is assumed that position is described by planes. For these reasons, it is assumed that interpolation for each MLS involves the recruitment of four independent or nonparallel planes.

The preceding description of planes has a physiological basis in the arm area of the motor cortex and somatosensory cortex. It was found that the discharge rate in primates increased as the hand moves toward planar surfaces in the Euclidean space. These planes were termed “position gradients” (Georgopoulos et al. 1984; Kettner et al. 1988). The discharge rate or tuning curve, of position gradients are modeled in this work as a normal distribution that is centered on a plane. In other words, the normal distance to a plane is modeled as the random variable. Note, a normal distribution was selected with the caveat that another bell‐shaped function might model neurophysiology of position gradients with greater accuracy.

**Figure 1:**
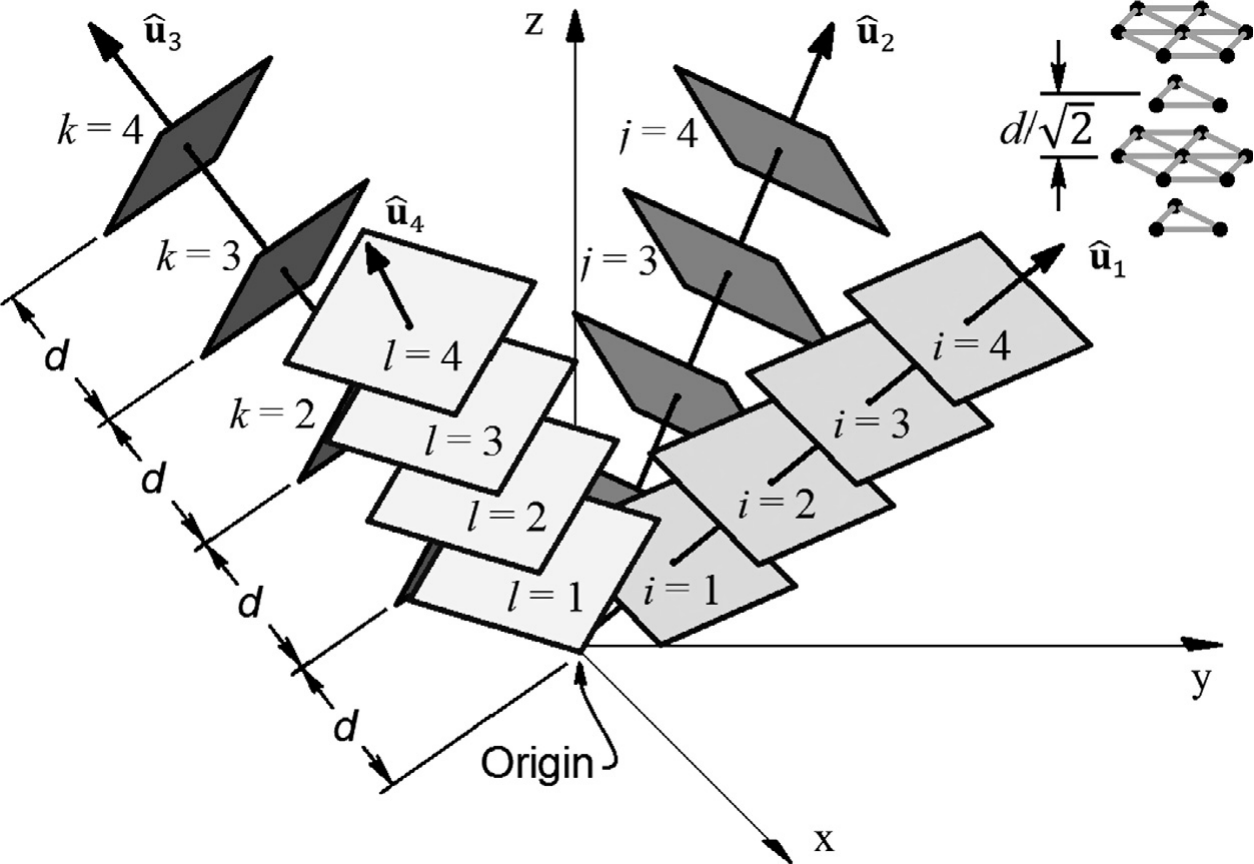
Plane geometry. All 4 sets of planes have the same plane spacing. The geometry of plane intersection points is depicted in the upper right.

The planes that describe position gradients are used to define a coordinate system. This coordinate system is partitioned using the mutual intersections of four sets of planes. The planes within each set of planes are all parallel and equally spaced from neighboring planes within that set. Four unit vectors are used to describe plane orientation and are given as follows,

(1) u^1=12^i+12^j+12^k

(2) u^2=−12^i+12^j+12^k

(3) u^3=12^i−12^j+12^k

(4) u^4=−12^i−12^j+12^k

where ^i, ^j, and ^k are unit vectors that point in the *x*,* y*, and *z* directions, respectively. The location of a given plane within each set of planes is given by an index. The distance between planes of consecutive indices is *d*, and the indices are given by *i*,* j*,* k*, and *l* for planes parallel to ${\hat{u}}_{1}$, ${\hat{u}}_{2}$, ${\hat{u}}_{3}$, and ${\hat{u}}_{4}$ respectively. A graphical depiction is provided in Figure 1.

The entire ensemble of plane intersections forms a grid of regularly spaced points in the Euclidean space. Note, the unit vectors given in equations* *((1), (2), (3), (4)) form a 45° angle with the *x*–*y* plane. However, the position gradient planes will continue to have mutual points of intersection for angles other than 45° and 45° was assumed ad hoc. The geometry of this grid of intersecting planes is revisited further on. Before continuing, a cautionary warning is in order. Specifying coordinates and indices using the planes described by equations** (1), (2), (3), (4) is cumbersome. This makes the mathematical description of position gradients appear complicated and unintuitive. However, outside of indexing, the underlying calculations are actually quite simple.

Ultimately, the position gradient arrangement in Figure 1 is used as a means to learn proprioception and to estimate inverse kinematics. Before embarking on those topics, the underlying model is described in equations** (5), (6), (7), (8), (9), (10), (11). The discharge rate or tuning curve, at distance *χ* from each plane is modeled by the normal distribution function given as follows,

(5) $$N\left(\text{mean}=0,\text{var}=c^{2}d^{2}\right)=\frac{1}{cd\sqrt{2\pi }}\text{exp}\left(-\frac{\chi^{2}}{2{\left(cd\right)}^{2}}\right)$$

where the “mean” value is assumed to equal zero and the “standard deviation” is assumed to equal *cd*. A plane spacing of *d *=* *40 mm and *c *=* *0.389, was used for all simulations. As is described latter on, the selection of *c* and *d* are related. A justification for these selections is provided in the Results section.

Let **p** denote the *observed* location of the hand. In terms of cortical processing, it is uncertain which specific anatomical feature of the arm or hand, plays a predominant role for positioning. For the purposes of simulation, hand positions are assumed to relate to the center of the wrist joint. Being able to identify which planes have the smallest normal distance from **p** is needed for future calculations. Along those lines, assume a 1 × 3 vector of Cartesian coordinates, given by **p**, denotes hand position. The plane indices that most closely neighbor **p** are given by the integers along the diagonal of the following,

(6) $$I\left(p\right)=\left[\begin{array}{cccc} i=\|p\cdot {\hat{u}}_{1}/d\| & 0 & 0 & 0\\ 0 & j=\|p\cdot {\hat{u}}_{2}/d\| & 0 & 0\\ 0 & 0 & k=\|p\cdot {\hat{u}}_{3}/d\| & 0\\ 0 & 0 & 0 & j=\|p\cdot {\hat{u}}_{4}/d\| \end{array}\right]$$

where the ‘|| ||’ operator rounds the dot products to the nearest integer. The projections of **p** onto the planes are given by the following equation.

(7) $$D\left(p\right)=\left[\begin{array}{cccc} p\cdot {\hat{u}}_{1} & 0 & 0 & 0\\ 0 & p\cdot {\hat{u}}_{2} & 0 & 0\\ 0 & 0 & p\cdot {\hat{u}}_{3} & 0\\ 0 & 0 & 0 & p\cdot {\hat{u}}_{4} \end{array}\right]$$

The distance from **p** to the nearest plane is determined by the following.

(8) Δ(p)=D(p)−dI(p)

Substituting Δ(p) for *χ* in *Eq.* (5) calculates the discharge rate at location **p** from each of the planes of the nearest virtual point. These discharge rates are given along the diagonal of the following matrix.

(9) $$\Omega \left(p\right)=\frac{1}{cd\sqrt{2\pi }}\text{exp}\left(-\frac{\Delta {\left(p\right)}^{2}}{2{\left(cd\right)}^{2}}\right)$$

Note, matrix Δ(p) is being squared, and raised to an exponential. These operations act individually on the elements for diagonal matrices such as this. Finally, the combined discharge rate, at location **p**, from all four planes is given as follows.

(10) R(p)=trace(Ω(p))

**Figure 2:**
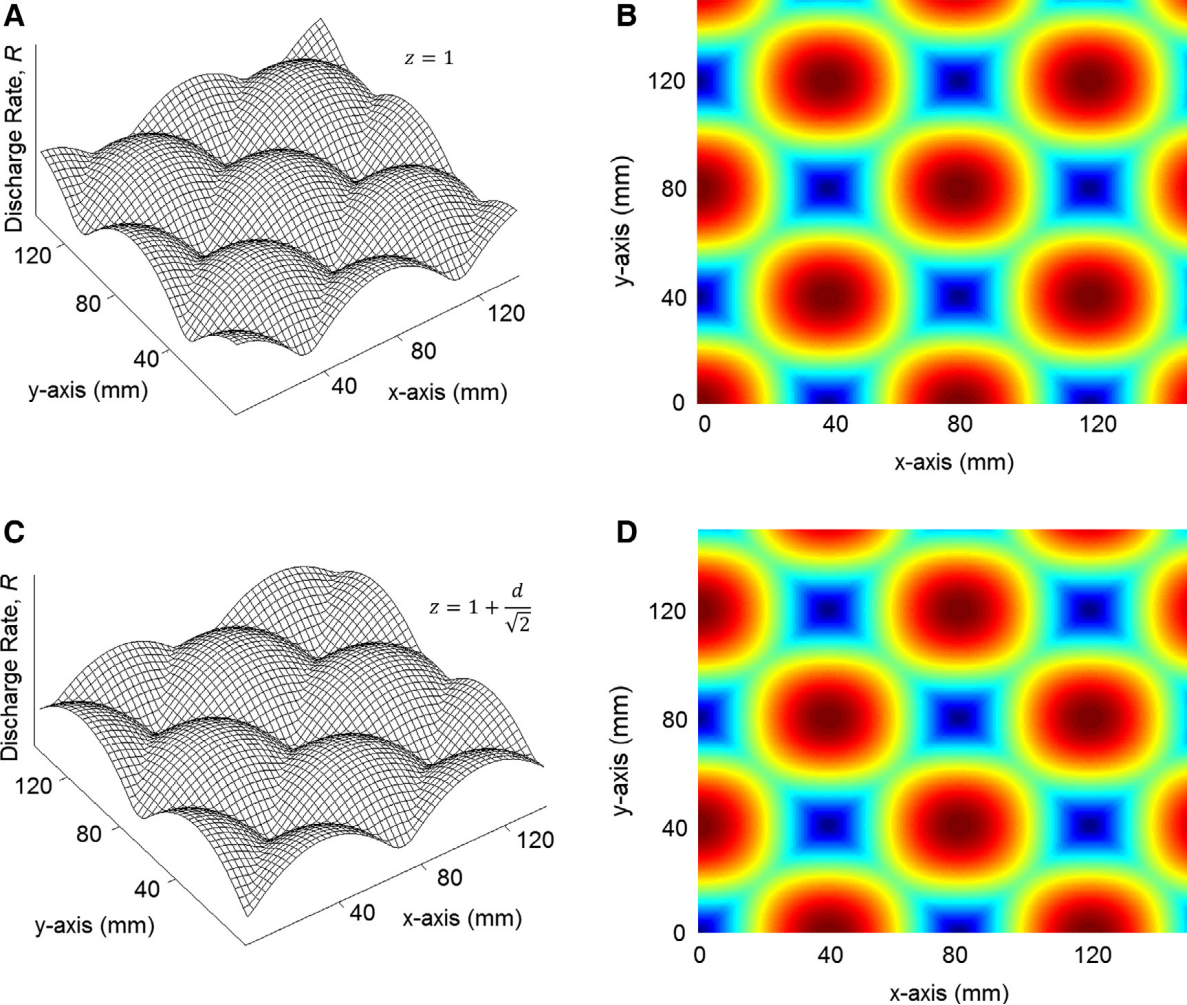
Discharge rate activity for movement along the *x*–*y* plane.

Because the planes in equation** (10) describe vector fields of increasing discharge rate as the hand approaches a given plane, the discharge rate, *R*, is especially high when the hand approaches locations where all four planes intersect. These points are referred to as “virtual points”. Though difficult to graph, the discharge rate around these points is analogous to the three‐dimensional probability density function or cloud that is associated with an electron orbiting a hydrogen atom. A grid of virtual points is depicted graphically in Figure 2 for two heights along the *z* axis. As the hand sweeps across the *x* and *y* axes, the discharge rate increases as the hand approaches the various virtual points. The discharge rate is depicted along the vertical axes in Figure 2A and C. It is depicted as a color map in (B) and (D).

Notice in Fig. 2 that levels of peak activity alternate for different distances along z. In particular, for the plane orientations given by equations** (1), (2), (3), (4), the virtual point locations toggle as *z* increment by *d*/$\sqrt{2}$. An executable script in MATLAB is provided in Appendix I. That script generates, and graphs this grid. For added perspective on the three‐dimensional structure, this virtual point grid geometry is very similar to the Bravais lattices found in hexagonal close packed crystals.

The contention that the motor cortex partitions space into families of equally spaced position gradients is a theoretical prediction and has not been reported experimentally. Notwithstanding, there is precedent for such an organization in the entorhinal cortex (Moser et al. 2008). It was found that “grid cells” exhibit patterns of activity that resemble Figure 2 for navigation tasks. Admittedly, grid cells were only reported for two‐dimensional navigation. However, it stands to reason that grid cells might also be at work for three‐dimensional navigation tasks such as tree climbing or flight, and that grid cell firing for planar movement might extend to three dimensions, as is the case here. A reasonable objection to the activity depicted in Figure 2 is that grid cell‐like activity has not been observed in the arm area of the cortex. Indeed, Figure 2 is not intended to depict position gradients, but rather, subcortical activity which is generated by a summation of position gradients. Equation** (10) is revisited when proprioceptive learning and adaption are considered. The predicted grid cell‐like activity in subcortical regions is considered further in the Discussion section.

The relationship between MLS's and position gradients now becomes easier to describe. Virtual points are cortical representations of points in external Euclidean space. When the hand is located at a virtual point, the set of associated muscle lengths constitutes an MLS given by

(11) $$s_{ijk}=\left(L_{1},L_{2},\ldots \;\;L_{9}\right)$$

where **s**_*i j k*_ ∈ **S**. For this work, six muscles in the elbow and two muscles in the shoulder are modeled. An anatomically fictitious ninth muscle is also included for reasons that are discussed later on. In this way, MLSs map points in Euclidean external space to muscle lengths in “muscle space”. Though, nine muscles are being simulated in this work, the hypothesis places no restrictions on the number of muscles being included in each MLS. The relationship between external space and muscle space is a few‐to‐many mapping; in that, more than one muscle is required to move a joint. Subscripts *i*,* j*, and *k* denote the index of the MLS within **S**. Indices *i*,* j*, and *k* have two interpretations. In equation** (6), they relate locations in Euclidean space using plane indices. In equation** (11), they index to sets of muscle lengths in **S**.

Consider what would happen if an arm MLS were somehow activated. The muscles of the arm should change in length such that the arm assumes a posture that moves the hand to the location of a virtual point. Of course, the musculature of the arm is viscoelastic and the arm would be unable to maintain the posture with perfect rigidity (Feldman and Latash 2004). Thus, displacing the hand from the virtual point would result in a restoring force. This restoring force should increase with increasing displacement. Indeed, such a phenomenon was observed using microstimulation of spinal cord gray matter in frogs (Bizzi et al. 1991; Giszter et al. 1993), rats (Tresch and Bizzi 1999), and cats (Lemay and Grill 2004). When spinal cord tissue was stimulated in these animals, their extremity would move to a repeatable location in space. When the extremity was displaced from one of these locations, the extremity exhibited a restoring force toward its point of attraction. This phenomenon was termed “convergent force fields” (Bizzi et al. 1991). Therefore, interneurons in the C3–C7 region of the spinal cord are possible candidates for storing arm MLSs. Locating MLSs closer to muscles (i.e., in the spinal cord as opposed to the cortex) also reduces latencies associated with muscle length error correction. This follows the same reasoning that explains why the stretch reflex or the nociceptive withdrawal reflex, are spinal phenomena (Andersen 2007).

### Virtual points hypothesis – inverse kinematic estimation

In principle, equation** (10) could specify a posture by increasing the neuronal activity for a given set of four planes. Peak discharge rate for the corresponding virtual point would then project onto an MLS in the spinal cord. However, the motor cortex must also perform interpolation between virtual points. In the following section, the mathematics of interpolation is described. Before embarking on a formal mathematical description, interpolation is described presently in words. Any desired posture will position the hand at a location that is neighbored by a collection of virtual points. Let **p**_D_ denote the *desired* location of the hand in Euclidean space. Note, **p**_D_ differs from **p;** in that, **p** relates the observed or actual hand location, while **p**_D_ relates to a target location. The position gradients of closer virtual points will exhibit larger discharge rates than more distant virtual points. Those discharge rates are used to weight the MLSs that correspond to the given virtual points. This makes sense because virtual points near **p**_D_ will map to an MLS that is more similar to the muscle lengths needed to achieve an arm posture that places the hand at **p**_D_. As such, they should receive a higher weight. Distant virtual points will map the MLSs that are less similar to the muscle lengths that achieve **p**_D_. As such, they should receive a lower weight.

The volley of descending weights or efferent activity, could be construed as the transmission of a “forward model” (Miall and Wolpert 1996). The scaled MLSs are then neurologically summed (Carandini and Heeger 1994) at lower levels to find a set of muscle lengths that achieve the interpolated posture. In other words, a collection of MLSs are activated simultaneously using various weights. Consistent with this theory, experimental evidence shows that simultaneous stimulation of several spinal cord sites results in a summative effect of convergent force fields (Tresch and Bizzi 1999).

The preceding remarks assume that efferent activity projects onto interneurons within the spinal cord. Descending weights are then used to excite neurons that encode for muscle lengths. Thereafter, motor neurons projecting from the ventral root are used to modulate skeletal muscle length. Presumably, spinal cord processes, such as the stretch reflex, in combination with muscle spindle afferent feedback, would act to eliminate the error between the specified muscle lengths and the actual muscle lengths. This error correcting process would persist until the muscles achieve the desired lengths in accordance with the equilibrium point hypothesis (Feldman 1986). Modeling of neuronal activity in spinal cord grey matter is outside the scope of this work.

Returning to the model, assume proprioception is learned. In other words, assume that the elements of **S** are populated with MLSs that accurately correspond to the muscle lengths of the arm when the hand is located at each virtual point. The vector between two virtual points is given by

(12) $$p_{R}\left(r,p,q\right)=\begin{array}{ccc} {}[rd & pd & qd/\sqrt{2}] \end{array}$$

where *p*,* q*, and *r*, are integers that describe relative indexing between virtual points. A derivation that describes the spacing between virtual points is given in Appendix II. The location of a virtual point relative to **p**_D_ is given by,

(13) $$p_{VP}\left(p_{D},r,p,q\right)=p_{D}+p_{R}\left(r,p,q\right)$$

where **p**_VP_ is a function of desired hand position and the relative indexing. Substituting equation** (13) into equation** (8) provides the distance from **p**_D_ to the planes of **p**_VP_ and is given by,

(14) $$\Delta \left(p_{D},r,p,q\right)=D\left(p_{D}\right)-dI\left(p_{VP}\left(p_{D},r,p,q\right)\right)$$

where **Δ** is a 4 × 4 diagonal matrix. Equation** (10) summed the discharge rates from the four closest position gradients or equivalently the closest virtual point. However, equation** (14) is being used for interpolation and more distant virtual points should contribute lower levels of activity than closer points. Recall that the position gradient discharge rate for each of the four planes of a given virtual point is given by the diagonal elements in equation** (9). As such, the minimum activity from **p**_D_ to the virtual point is found by expressing equation** (9) as a function of equation** (14) and in finding the minimum value among the diagonal elements. This is accomplished as follows.

(15) $$\omega \left(p_{D},r,p,q\right)=\text{min}\left(\Omega {\left(p_{D},r,p,q\right)}_{11},\ldots \;,\Omega {\left(p_{D},r,p,q\right)}_{44}\right)$$

Note, equation** (15) is a scalar, not a matrix as was the case for equation** (9). Recall in an earlier discussion that interpolation within a volume requires four independent pieces of information. For this reason, four planes with different orientations were included in this model. The need for four nonparallel planes is reflected in equation** (15). For perspective, interpolation was first tried using only three planes. Interpolation must consider the nearest planes that surround **p**_D_. If this is done using only three plane orientations, then interpolation must include two planes with the same orientations and consecutive indices. In that case, the volume that surrounds **p**_D_ is a sliver‐shaped polyhedron with a distant virtual point as one of its vertices. Recall that virtual points are being used to perform interpolation from the Euclidian space to muscle space. For nonlinear interpolations such as this, the reference points or virtual points, should be as close **p**_D_ as possible. For a sliver‐shaped polyhedron, the distant virtual point is equally likely to be included in the interpolation as the closer points. This has the effect of increasing the error to such an extent that interpolation is no more accurate than simply selecting the nearest virtual point. It is for this reason that four different plane orientations were required.

The discharge rate given by equation** (15) is used as part of a weighed arithmetic mean that includes a collection of virtual points that neighbor **p**_D_. More specifically, interpolation will make use of the 3^3^ = 27 neighboring virtual points in the vicinity of **p**_D_. The scaling factor for each virtual point is calculated as follows.

(16) $$\hat{\omega }\;\left(p_{D},r,p,q\right)=\frac{\omega (p_{D},r,p,q)}{\sum_{r=-1}^{1}\sum_{p=-1}^{1}\sum_{q=-1}^{1}\omega \left(p_{D},r,p,q\right)}$$

The indices of the MLSs in **S** that are scaled by equation** (16) are calculated using diagonal elements within the following 4 × 4 matrix.

(17) $$\eta \left(p_{D},r,p,q\right)=I\left(p_{VP}\left(p_{D},r,p,q\right)\right)$$

Finally, the muscle lengths in **S** are interpolated as a function of **p**_D_ using equation** (17) for the indices of **s** as follows,

(18) LpD=∑r=−11∑p=−11∑q=−11^ωpD,r,p,qsη11η22η33

where **L** is a 1 × 9 vector of interpolated muscle lengths. Note, indexing to a coordinate only requires 3 planes. Therefore, the subscript η_44_ is omitted from equation** (18). Beyond the complexities associated with indexing, equation** (18) is essentially a simple equation that sums muscle lengths by a weighted arithmetic mean of nearby position gradients.

### Virtual points hypothesis – proprioceptive learning/adaption

Animals must continually adapt to changing kinematics as they mature and grow. Having described virtual points and their relationship with MLSs, a learning/adaption process is describable. As the hand moves through space, the hand will inevitably pass close to virtual points. Let **s**(**p**) denote the muscle lengths for the hand's current position. The location of the hand is discernable from visual or tactile observation, and this will result in some level of discharge rate given by equation** (10). If the hand moves near a virtual point, the associated muscle lengths constitute a fairly accurate MLS solution for that location. If **s**(**p**) happens to map the hand closer to the virtual point than the formerly learned MLS, then the MLS should be updated with the better solution. In this way, the CNS is constantly checking and updating **S**. This process is now stated mathematically. Recall that the first three diagonal elements in equation** (6) provide the indices of the nearest virtual point to **p**. Learning is thus accomplished using the following condition statement.

(19) $$s_{I{\left(p\right)}_{1},I{\left(p\right)}_{2},I{\left(p\right)}_{3}}=\left\{\begin{array}{c} s(p),ifR\left(p\right)>R\left(p_{I{\left(p\right)}_{1},I{\left(p\right)}_{2},I{\left(p\right)}_{3}}\right)\\ s_{I{\left(p\right)}_{1},I{\left(p\right)}_{2},I{\left(p\right)}_{3}},\text{otherwise}. \end{array}\right.$$

The update process in equation** (19) suggests that MLS's are retained and updated. If MLS's were retained in the spinal cord, this in turn suggests a spinal learning mechanism that is mediated by higher cortical levels (Kargo and Giszter 2000; Cheung et al. 2009; Roh et al. 2011). A number of experiments have demonstrated spinal motor leaning mechanisms, that is, neuroplasticity, (Chen and Wolpaw 2002; Wolpaw and Chen 2006; Edgerton et al. 2005) and subcortical motor learning has been proposed as a mechanism in stroke recovery (Simkins et al. 2014).

The inequality in equation** (19) evaluates if the discharge rate of the current hand position is greater than the discharge rate that would occur if the hand were positioned using the previously stored MLS. In doing this comparison, equation** (19) suggests that the CNS calculates position gradients from MLSs, presumably in the reverse process of equation** (18). While this assumption was used for the purposes of simulation, there are alternative, and more plausible means for updating **S**. This update process essentially reduces to a problem of retaining information based on an elevated discharge rate. In this case, the retained information are muscle lengths. The elevated discharge rate result from cortical activity associated with equation** (10). From an algorithmic perspective, learning processes such as this are used widely in artificial neural networks and there is a substantial body of relevant work in reinforcement learning (Kaelbling et al. 1996). A physiologically relevant model would leverage work done using long‐term potentiation. Indeed, long‐term potentiation models have a long history and numerous models have been proposed since long‐term potentiation was confirmed experimentally in the hippocampus (Bliss and Collingridge 1993). Thus, inclusion of a learning model in the spinal cord would largely duplicate past work. For this reason, equation** (19) was deemed a sufficient simplification for these purposes.

This model includes two distinct mechanisms. Notice that equation** (19) is a function of the actual hand position, **p**, not the desired position, **p**_D_. This distinction relates to the fact that actual hand positions, or even trajectories, may differ from what is desired (Bizzi et al. 1983; Won and Hogan 1995). For example, if the desired position was behind a fixed obstacle, the hand might simply push against the obstacle (Bizzi et al. 1992). If a desired trajectory was interrupted by an unexpected force field, the field might push the hand along a different trajectory (Shadmehr and Musa‐Ivaldi 1994; Mussa‐Ivaldi and Patton 2000). One might say that equation** (18) relates to where the hand should go while equation** (19) relates to where the hand is. Estimating where the hand should go relates to movement planning and efferent activity. Evaluating where the hand is relates to learning and reafferent activity. Thus, even though equations** (18) and (19) utilize similar mechanisms, the two calculations are essentially different. This distinction is revisited in the Discussion section.

### Computer simulation

The simulation is divided into a “learning” phase and an “adaption” phase. During learning, the CNS learns to position an adult sized arm from an empty **S** matrix, that is, from a “blank slate”. During the adaption phase, the CNS adjusts a previously populated **S** matrix to adapt to changing kinematics that is associated with limb growth.

In the learning phase, the **S** matrix is initially populated with zeros. Learning is accomplished by populating **S** with MLSs according to equation** (19) using randomly generated movements, similar to a Monte Carlo method. One billion randomly generated joint angles, or “iterations” were performed. An analogy of this learning phase is of an infant flailing its arms at random. Let FK(**s**_*ijk*_) denote the forward kinematic solution, or position, that the hand achieves when the muscles are stretched to the lengths given by **s**_*ijk*_. Recall that equation** (12) described the relative location of a virtual point, **p**_**R**_, using indices *p*,* q*, and *r*. However, expressing equation** (12) as a function of *i*,* j*, and *k* describes the Cartesian coordinate of a virtual point relative to the origin, see Figure 1. The average distance between FK(**s**_*ijk*_) and virtual point locations, **p**_**R**_(*i*,*j*,*k*), are given by

(20) ^dlearn=1/μ∑m=1μ‖FK(sijk)−pR(i,j,k)‖

where *μ* is the number of elements in **S** with nonzero MLSs. As the hand moves early in the learning process, it is unlikely that the hand will have traveled close to most virtual points and ${\bar{d}}_{\mathrm{learn}}$ will be large. As the hand continues to move randomly, it will have more opportunities to pass nearer to virtual points and ${\bar{d}}_{\mathrm{learn}}$ will diminish in size. Thus, equation** (20) is a measure of the completeness of proprioceptive learning.

Demonstrating the adaption phase is accomplished by increasing the length of *L*_u_ and *L*_f_ by five discrete lengths. For each growth size, the arm is then moved at random as if the arm performed numerous, unforeseen movements associated with activities of daily living. In this way, equation** (19) adjusts **S** to new kinematics. Growth is simulated as if the upper arm and forearm were lengthened instantaneously by 1, 2, 3, 4, or 5 mm. Thus, the overall arm length (upper arm plus forearm) increases by 2, 4, 6, 8, and 10 mm, respectively. A test trajectory is then used to evaluate the position errors caused by growth. Of course, the notion that the overall arm length might grow instantaneously, or “overnight,” by 10 mm, or even 2 mm, is implausible. The purpose of modeling proprioceptive adaption is to demonstrate the ability of equation** (19) to adapt to changing kinematics. In reality, this process would occur imperceptivity during maturation as the limbs grow slowly over time.

The test trajectory consists of 140 desired points, (**p**_D_'s), or “via points”. Thus, a series of hand positions was used to simulate hand trajectories, or “movement from posture” (Merton 1953). The via points forms a 200 mm by 80 mm rectangle. The average error for all 140 points along this test trajectory was calculated by following equation.

(21) $$\bar{\text{error}}=\frac{1}{140}\sum_{m=1}^{140}\;\|p_{m}-p_{Dm}\|$$

where *m* is the *m*th via point along the test trajectory.

A two‐joint simulation of the arm was implemented in MATLAB (Mathworks Inc., Natick, MA, USA). Hand positioning was simulated using the following forward kinematic model,

(22) $$x\left(\theta_{1},\theta_{2}\right)=L_{u}\;\text{cos}\left(\theta_{1}\right)+L_{f}\;\text{cos}\left(\theta_{1}+\theta_{2}\right)$$

(23) $$y\left(\theta_{1},\theta_{2}\right)=L_{u}\;\text{sin}\left(\theta_{1}\right)+L_{f}\;\text{sin}\left(\theta_{1}+\theta_{2}\right)-600$$

(24) $$z\left(\theta_{3}\right)=\theta_{3}$$

where *θ*_1_ is the shoulder angle, *θ*_2_ is the elbow angle, *L*_u_ is the upper arm length of 361 mm, and *L*_f_ is the forearm length of 278 mm. These lengths match the average length for an adult man (Winter 2005). The purpose of *θ*_3_ is discussed later on.

There is an important point worth mentioning for equations** (22) and (23). These are forward kinematic solutions that map joint angles to Cartesian coordinates. They are used for simulation purposes. Inverse kinematics calculates joint angles, or by extension, muscle lengths, as a function of Euclidian coordinates. The inverse problem is much more difficult to calculate than the forward problem. For a first hand perspective, consider solving for *θ*_1_(*x*,* y*) and *θ*_2_(*x*,* y*) using equations** (22) and (23). Despite their simple appearance, the inverse solution is not easily obtainable. Solving for inverse kinematics often requires geometric reasoning, algebraic substitutions, and trigonometric identities. In many cases, there is no inverse solution and iterative techniques, such as Newton's method, are required. An inverse kinematic solution for equations** (22) and (23) is never used in this work. Instead, inverse kinematics is estimated using position gradients and MLSs exclusively. Demonstrating a way for the CNS to perform such complicated calculations by leveraging known physiology is core to this work.

**Figure 3:**
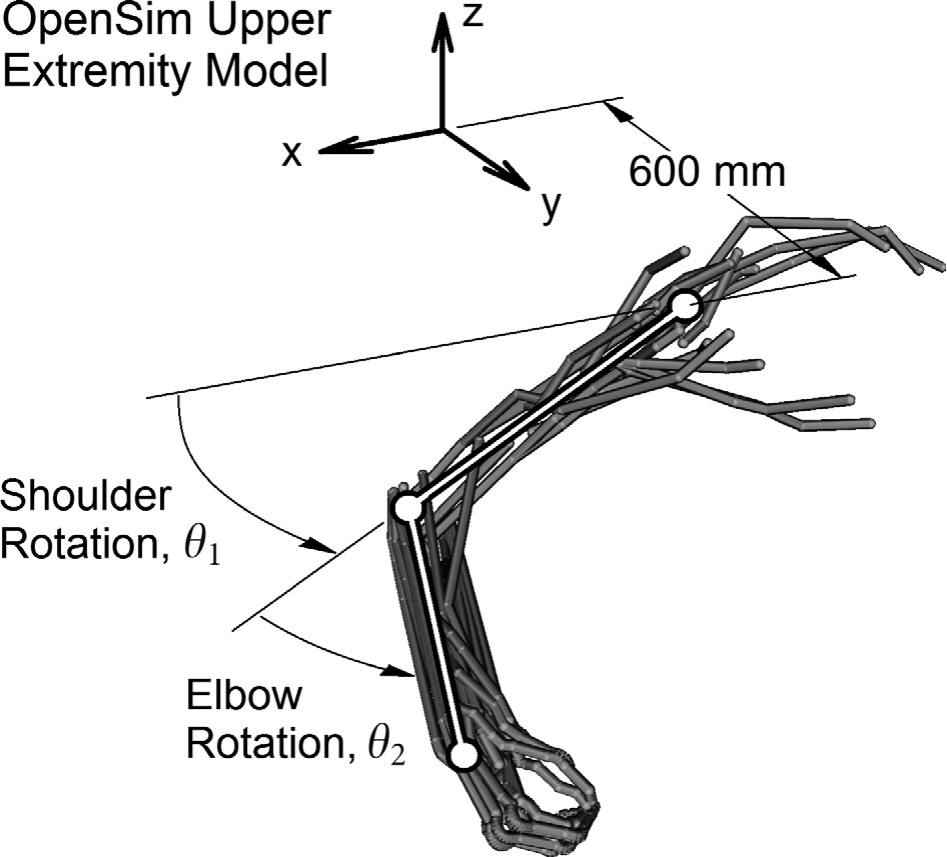
The OpenSim muscle model. The origin of the torso coordinate frame is located behind the shoulder.

Muscles are being simulated, not joint angles. Therefore, joint angles in equations** (22) and (23)need to be converted into muscle lengths. Muscle lengths were estimated as a function of joint angles using OpenSim 3.0 (Stanford University, Palo Alto, CA; Delp et al. 2007), in conjunction with an upper limb muscle model (Holzbaur et al. 2005). Joint angles ranged from 23° to −90° for *θ*_1_, and 3° to 130° for *θ*_2_. These angles were selected such that the elbow and wrist were constrained to move in a sectional plane that intersects the glenohumeral joint, see Figure 3 (Mussa‐Ivaldi 1997). Curve fits were then applied to the simulated muscle lengths using a second‐order polynomial. The muscle names, abbreviations, curve fits, and goodness‐of‐fit (*R* ^2^) are given in Table 1. This nine muscle model includes three agonist and three antagonist muscles pairs for the elbow, as well as an agonist and antagonist muscle for the shoulder.

**Table 1:** Summary of the muscle model

Muscle	Abbreviation	2nd Curve fit	*R* ^2^
Shoulder			
Anterior Deltoid	DELT1	L_1_ = −7e‐4*θ*_1_^2^ − 0.6*θ*_1_ + 102.6	0.99
Posterior Deltoid	DELT3	L_2_ = −1e‐3*θ*_1_^2^ + 0.7*θ*_1_ + 121	0.98
Elbow			
Triceps Long Head	TRIlong	L_3_ = −1e‐3*θ*_2_^2^ + 1.1*θ*_2_ + 100	>0.99
Triceps Medial Head	TRImed	L_4_ = 3e‐4*θ*_2_^2^ + 0.3*θ*_2_ + 68.2	0.99
Triceps Lateral Head	TRIlat	L_5_ = 2e‐3*θ*_2_^2^ + 0.5*θ*_2_ + 71.4	0.99
Biceps Brachii (long head)	BIClong	L_6_ = −5e‐3*θ*_2_^2^ − 6e‐2*θ*_2_ + 166	0.99
Biceps Brachii (short head)	BICshort	L_7_ = −5e‐3*θ*_2_^2^ + 0.1*θ*_2_ + 197.4	0.97
Brachiali	BRD	L_8_ = −8e‐3*θ*_2_^2^ − 0.2 *θ*_2_ + 194.4	0.99
Fictitious	Z	L_9_ = *θ*_3_	1.0

The actual functional relationship between muscle length and joint angles are certainly more complicated than is suggested by second‐order polynomials. However, the purpose of simulating the nonlinear relationships in Table 1 is to show that the model can learn and adapt to nonlinear muscle kinematics. As is evident from the *R* ^2^ values in Table 1, the lowest *R* ^2^ is 98%. Therefore, the actual kinematic equations would differ only slightly from these second‐order approximations. Another approximation in Table 1 relates to independence. Specifically, BIClong, BICshort, and TRIlong actually change in length for both elbow rotation and shoulder rotation. However, they are modeled as only changing in length in response to elbow flexion and extension.

An anatomically fictitious muscle is included in equation (24), and in Table 1 as muscle Z. Z varies 1:1 with *θ*_3_. Not only is prismatic translation anatomically incorrect but also the Z muscle has no antagonist. Inclusion of Z relates to the fact that the four plane orientations given by equations (1), (2), (3), (4) establish a coordinate system that is intrinsically three dimensional. For example, consider the test trajectory. The test trajectory is constrained to a plane that is parallel to the *x*–*y* plane (not to be confused with position gradient planes). However, the virtual points used for interpolation reside above and below the trajectory plane. Those virtual points must have nonzero elements in **S**. In order to populate those MLSs in **S**, the hand must be allowed to traverse space in the z direction during random movements. Therefore, **S** was populated by allowing Z to translate though a distance of $6d/\sqrt{2}\approx 170\;mm$.

## Results

**Figure 4:**
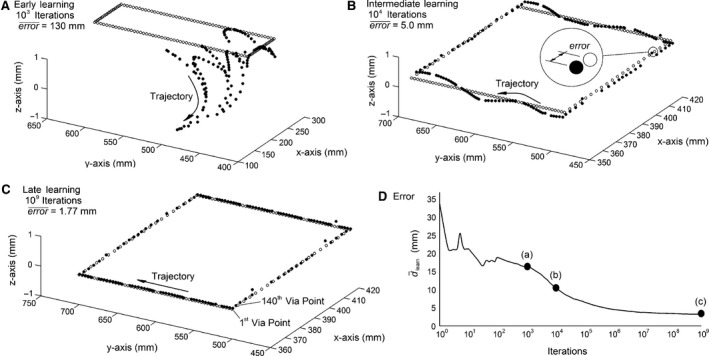
Parts A–C depict the rectangular test trajectory that is generated during various stages of learning. Note, (A) is rescaled but the trajectory is identical to (B) and (C). Depicted in (D) is the average distance between leaned solutions and virtual points.

Depicted in Figure 4A–C are three test trajectories at various stages in the learning process. Hollow circles are target points, **p**_D_, and solid point indicate the position of the hand, **p**, using the interpolated muscle lengths. After only 10^3^ iterations, set **S** is insufficiently learned and equation (18) is unable to accurately interpolate muscle lengths. Accordingly, $\bar{\mathrm{error}}$ = 130 mm, see (A). By 10^9^ iterations, **p**_D_ and **p** have more overlap and $\bar{\mathrm{error}}$ drops to only 1.77 mm, see (C). Thus, Figure 4A–C demonstrates the ability of equation (18) to interpolate muscle lengths. It also demonstrates the ability of equation (18) to learn kinematics starting with a matrix of zeros.

Depicted in Figure 4D is a plot of ${\bar{d}}_{\mathrm{learn}}$versus the number of iterations. As the number of iterations continues beyond 10^9^, ${\bar{d}}_{\mathrm{learn}}$ is expected to continue approaching zero. Accordingly, the approximations given by equation (18) would continue to improve, albeit slightly. The relationship between ${\bar{d}}_{\mathrm{learn}}$ and $\bar{\mathrm{error}}$ is apparent from the (A), (B), and (C) reference points included in (D).

By 10^9^ iterations, ${\bar{d}}_{\mathrm{learn}}$ is 3.21 mm, see Figure 4D. Though this number of iterations might appear large, it is not unreasonable. This computer simulation is inherently discrete. Each iteration simulates the hand moving instantaneously from one point to another. In reality, the hand traverses space in a continuous manner. To justify this level of iteration, let us consider the likelihood of achieving a ${\bar{d}}_{\mathrm{learn}}$ = 3.21 mm using a continuous trajectory. The geometry of virtual points described by equation (12) is somewhat complicated. As a rough approximation, assume that the hand randomly sweeps across space and that the distance between virtual points is *d*. As such, the hand‐to‐virtual point proximity will range from 0 (hand is incident on the virtual point) to *d*/2 (hand is in between two virtual points). The probability that the hand would pass a virtual point with a proximity of between 0 and 3.21 mm is given by 3.21/(0.5*d*) = 0.16. If it is assumed that the distance between virtual points is approximately *d*, then for every meter that the hand travels, it sweeps past 1/*d *=* *25 virtual points. For every meter that the hand travels, it will near roughly 0.16 × 25 = 4 virtual points with a proximity ≤ 3.21 mm. Therefore, ${\bar{d}}_{\mathrm{learn}}$ seems reasonable although 10^9^ iterations were required using simulation.

Recall that equation (5) included *c *=* *0.389 and *d *=* *40. A justification for these constants is now provided. Recall that the tuning curve was modeled with a normal distribution. Increasing *c* effectively spreads the position gradient activity across a larger volume. In other words, *cd* in equation (9) substitutes for the “standard deviation,” or *σ*. Constant *c* was obtained iteratively by minimizing $\bar{\mathrm{error}}$. Constant *c* equal to 0.389 optimally reduces $\bar{\mathrm{error}}$ independently of *d*. From the perspective of a sensitivity analysis, $\bar{\mathrm{error}}$ doubles when *c* increases by 50% or decreases by 15%. Therefore, interpolation in equation (18) depends critically on *c*. This also suggests that interpolation predominantly involves virtual points in the immediate vicinity of **p**_D_. Note, equation (19) was comparatively insensitive to *c* in that variations in *c* had no noticeable effect on proprioceptive learning or adaption.

These considerations beg the question as to why a plane spacing of *d *=* *40 mm was selected. Using a plane spacing of 40 mm with minimal error translates to an *σ *= 15.56 mm. Being derived from a normal distribution, this means that the 95% of the position gradient discharge rate activity would occur within 4*σ*, or approximately 62 mm. While there is no known model that describes position gradients in exactly the same way that they are described in this work, graphical depictions of position gradient data was provided in (Kettner et al. 1988). In that work, position gradient activity was presented within a 152 mm cubic workspace. The 62 mm wide swath of activity that is assumed in this work approximately matches the swatch of activity depicted in (Kettner et al. 1988).

**Figure 5:**
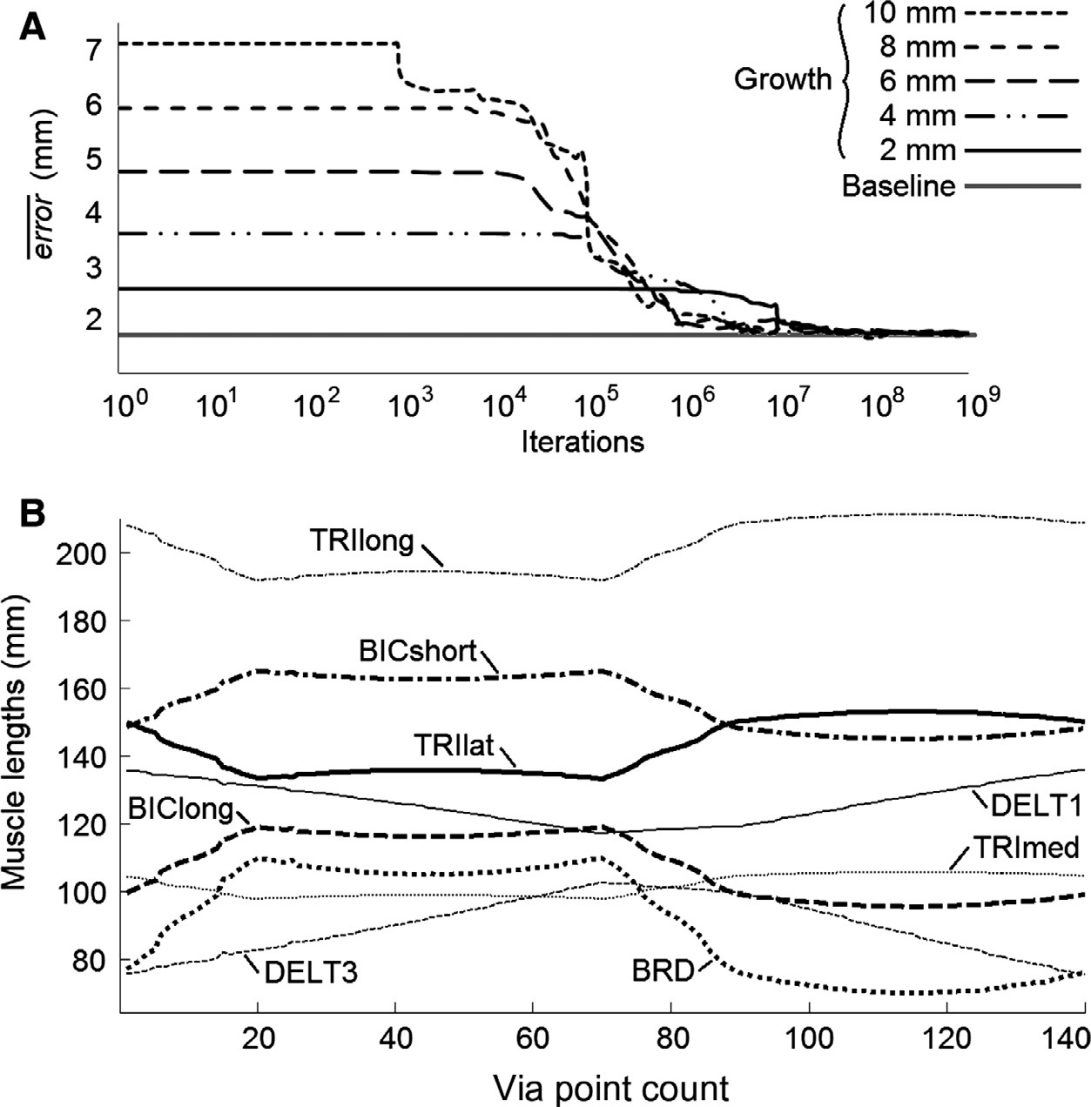
Proprioceptive adaption is summarized in (A). Depicted in (B) are the interpolated muscle lengths that guide the hand through the test trajectory.

Proprioceptive adaption is depicted in Figure 5. The numbers of iterations are depicted along a logarithmic scale. Again, the plots in Figure 5A depict proprioceptive adaption after the upper arm and forearm are each lengthened by 1, 2, 3, 4, or 5 mm. For the first 10^3^–10^5^ iterations, the hand moves using an **S** matrix that was learned with the pregrowth kinematics. As such, the new kinematics is not yet learned and the growth causes an error that is proportional to the growth. By 10^9^ iterations, equation (19) has updated **S** to account for the growth and the $\bar{\mathrm{error}}$ is comparable to the pregrowth baseline $\bar{\mathrm{error}}$ of 1.77 mm. Again, even though these levels of growth are exaggerated, they demonstrate the ability of equation (19) to adapt to changing kinematics through maturation.

The interpolated muscle lengths after 10^9^ iterations are depicted in Figure 5B. These muscle lengths guide the hand to via points plotted in Figure 4C. Though the muscle lengths in Figure 5B are plotted as continuous lines, they are actually discrete sets of muscle lengths for each via point. Ordinarily, such calculations would require inverse kinematic solutions. The results depicted in Figure 5B are of particular importance because they graphically demonstrate the ability of equation (18) to calculate muscle lengths. In other words, Figure 5B demonstrates the ability of equation 18 to estimate inverse kinematics in muscle space given desired points in Euclidian space.

## Discussion

Position gradients and convergent force fields were exploited in this work. Through computer simulation, it was shown that these physiological mechanisms can be structured in a way to learn kinematics, select muscle lengths in order to position the hand as desired, and to adapt to changing kinematics through maturation. Fundamentally, the hypothesis is simple. Muscle lengths are calculated using a weighted sum of MLSs. Learning was accomplished using a simplified condition statement involving those weights. This construction, if true, helps to bridge the gap between low‐level muscle enervations in muscle space with high‐level cortical representations of positioning in the Euclidian space. Moreover, position gradients were modeled as tuning curves. In turn, tuning curves were used as a means of spatial interpolation for the hand. The resulting patterns of activity resemble patterns of activity observed in grid cells. An analogous mechanism might be at work for spatial navigation tasks involving grid cells. Given that tuning curves are also observed in connection to auditory and visual processing, tuning curves may provide a similar function for auditory and visual interpolation.

Even though this model was constructed from experimentally determined findings from the literature, several key assumptions were made. The position gradients that were modeled as planes used orientations and plane spacing that were selected ad hoc. Despite the fact position gradients were found in animals, the various orientations that they assume are unknown. With respect to plane spacing, the simulated distances were 40 mm. A smaller plane spacing would result in more virtual points, thereby increasing the information storage demands on the CNS. However, this would provide the benefit of reducing position error. Experimentally determined proprioceptive position errors are significantly larger than the 1.77 mm average error achieved in this work (Van Beers et al. 1998). Therefore, plane spacing much less than 40 mm would seem unlikely.

This model makes several specific predictions about position gradients. First, for a given limb, there should be a minimum of four different position gradient orientations. Second, position gradients of a given orientation should repeat with equal spacing. Third, there should exist locations where four planes, of different orientation, converge at a common point. These are theoretically derived predictions. Given that the location and orientation of position gradients are measurable, these predictions are not only specific, they are falsifiable.

As was alluded to in the Methods section, position gradients are used in two distinct ways. Equation (18) uses position gradients to weight MLSs as a means of interpolation. That sort of calculation lends itself to motor planning and the generation of efferent commands. Equation (19) uses position gradients to update MLSs as a means of achieving proprioceptive learning. That sort of calculation lends itself to reafferent processing. Accordingly, position gradients were observed in both the motor cortex, and area 5 for somatosensory association (Ashe and Georgopoulos 1994). Given that the motor cortex is associated with dispatching efferent information, and area 5 is associated with processing reafferent information (Georgopoulos et al. 1984), it stands to reason that equation (18) would model activity in the motor cortex while equation (19) models activity in area 5. Assuming that MLSs reside in subcortical structures, possibly in interneurons of the spinal cord, the hypothesis predicts that area 5 should have projections along the spinal cord whereby MLSs are continually evaluated and updated. Recall that Figure 2 depicted levels of activity that were allegedly similar to grid cell behavior. A fourth, and final prediction is that descending commands to MLSs should exhibit grid cell‐like activity. More specifically, for each nerve fiber that projects onto an MLS, equation (18) should generate increasing levels of activity as the hand nears the associated virtual point. Assuming that MLSs are retained within spinal grey matter, this activity should be present along descending pathways within the spinal cord.

## Conflict of Interest

None declared.

## Appendix I

This MATLAB script generates the plot in Figure 2B and D using equation (10). The volume under the plot depicts a convolution of discharge rate along the *x*–*y* plane.

*z* = 1;

*c* = 0.389;

*d* = 40; %Distance between planes

*U* = [1/2 1/2 1/sqrt(2); −1/2 1/2 1/sqrt(2);1/2 −1/2 1/sqrt(2); …

−1/2 −1/2 1/sqrt(2)];%Plane directions as unit vector

inc = 0.3;%Set to 1.0 for accurate axis scaling.

for *i* = 1:500

for *j* = 1:500

impulses = 0; %Represents action potential rate

P = [*i**inc,*j**inc,*z*];

for *k* = 1:4;

D = [dot(P,U(1,:)) dot(P,U(2,:)) dot(P,U(3,:))…

dot(P,U(4,:))];

I = round(D/*d*); %Plane index

for *m* = 1:4

W = 1/(*d***c**sqrt(2*pi()))*exp(−(D(*m*)…

−I(*m*)**d*)^2/(2*(*c***d*)^2));

Impulses = impulses + W;

end

end

IMPs(*i*,*j*) = impulses;

end

end

axis(‘off’), surface(IMPs, ‘EdgeColor’, ‘none’)

## Appendix II

Consider the plane orientation given by equations (1), (2), (3), (4). For each index where *i *= *j *= *k *= *l*, all four planes have a common intercepts at the *x*,* y*, and *z* axes. Given that each unit vector forms a 45° angle with the *x*–*y*, plane it follows that the triangle formed by the unit vector, the axes, and the plane forms a 45° right triangle. Given that the hypotenuse of these triangle are length *d* times the index, it follows that the intercepts are at the product of the index, the plane distance, and the $\sqrt{2}$. Given these intercepts, equation (1) is rewritten as equations of a plane as follows,

(A1) $$\frac{1}{2}\left(x_{1}=0\right)+\frac{1}{2}\left(y_{1}=0\right)+\frac{1}{\sqrt{2}}\left(z_{1}=\sqrt{2}di\right)+C=0$$

where subscript 1 indicates that this is the plane equation for (1). Solving for C in equation A1 we find that C = −*di*. By similar reasoning, the plane equations for (1), (2), (3) are

(A2) $$\frac{1}{2}x_{1}+\frac{1}{2}y_{1}+\frac{1}{\sqrt{2}}z_{1}-di=0$$

(A3) $$-\frac{1}{2}x_{2}+\frac{1}{2}y_{2}+\frac{1}{\sqrt{2}}z_{2}-dj=0$$

(A4) $$\frac{1}{2}x_{3}-\frac{1}{2}y_{3}+\frac{1}{\sqrt{2}}z_{3}-dk=0$$

where subscripts 2 and 3 indicate that these are the plane equations for equations (2) and (3), respectively. For these plane equations to mutually intersect at virtual points, the planes must be independent. In other words, the four family of planes must have different orientations. Accordingly, the location of virtual points occurs when *x*_1_ = *x*_2_ = *x*_3_, *y*_1_ = *y*_2_ = *y*_3_, and *z*_1_ = *z*_2_ = *z*_3_.This system of plane equations is expressed as follows,

(A5) $$\left[\begin{array}{c} di\\ dj\\ dk \end{array}\right]=\left[\begin{array}{ccc} \frac{1}{2} & \frac{1}{2} & \frac{1}{\sqrt{2}}\\ -\frac{1}{2} & \frac{1}{2} & \frac{1}{\sqrt{2}}\\ \frac{1}{2} & -\frac{1}{2} & \frac{1}{\sqrt{2}} \end{array}\right]\left[\begin{array}{c} x_{ijk}\\ y_{ijk}\\ z_{ijk} \end{array}\right]$$

Given that all three planes are nonparallel, (A5) is full rank. Solving simultaneously obtains the following,

(A6) $$x_{ijk}=d\left(i-j\right)$$

(A7) $$y_{ijk}=d\left(i-k\right)$$

(A8) $$z_{ijk}=\frac{d}{\sqrt{2}}\left(j+k\right)$$
